# Mismatch Repair Balances Leading and Lagging Strand DNA Replication Fidelity

**DOI:** 10.1371/journal.pgen.1003016

**Published:** 2012-10-11

**Authors:** Scott A. Lujan, Jessica S. Williams, Zachary F. Pursell, Amy A. Abdulovic-Cui, Alan B. Clark, Stephanie A. Nick McElhinny, Thomas A. Kunkel

**Affiliations:** 1 Laboratory of Molecular Genetics and Laboratory of Structural Biology, National Institute of Environmental Health Sciences, Research Triangle Park, North Carolina, United States of America; 2 Department of Biochemistry, Tulane University, New Orleans, Louisiana, United States of America; 3 Department of Biology, Augusta State University, Augusta, Georgia, United States of America; 4 United States Army Research Office, Research Triangle Park, North Carolina, United States of America; The Hospital for Sick Children and University of Toronto, Canada

## Abstract

The two DNA strands of the nuclear genome are replicated asymmetrically using three DNA polymerases, α, δ, and ε. Current evidence suggests that DNA polymerase ε (Pol ε) is the primary leading strand replicase, whereas Pols α and δ primarily perform lagging strand replication. The fact that these polymerases differ in fidelity and error specificity is interesting in light of the fact that the stability of the nuclear genome depends in part on the ability of mismatch repair (MMR) to correct different mismatches generated in different contexts during replication. Here we provide the first comparison, to our knowledge, of the efficiency of MMR of leading and lagging strand replication errors. We first use the strand-biased ribonucleotide incorporation propensity of a Pol ε mutator variant to confirm that Pol ε is the primary leading strand replicase in *Saccharomyces cerevisiae*. We then use polymerase-specific error signatures to show that MMR efficiency *in vivo* strongly depends on the polymerase, the mismatch composition, and the location of the mismatch. An extreme case of variation by location is a T-T mismatch that is refractory to MMR. This mismatch is flanked by an AT-rich triplet repeat sequence that, when interrupted, restores MMR to >95% efficiency. Thus this natural DNA sequence suppresses MMR, placing a nearby base pair at high risk of mutation due to leading strand replication infidelity. We find that, overall, MMR most efficiently corrects the most potentially deleterious errors (indels) and then the most common substitution mismatches. In combination with earlier studies, the results suggest that significant differences exist in the generation and repair of Pol α, δ, and ε replication errors, but in a generally complementary manner that results in high-fidelity replication of both DNA strands of the yeast nuclear genome.

## Author Summary

The stability of complex and highly organized nuclear genomes partly depends on the ability of mismatch repair (MMR) to correct a variety of different mismatches generated as the leading and lagging strand templates are copied by three polymerases, each with different fidelity. Here we provide the first comparison, to our knowledge, of the efficiency of MMR of leading and lagging strand replication errors. We first confirm that Pol ε is the primary leading strand replicase, complementing earlier assignment of Pols α and δ as the primary lagging strand replicases. We then show that MMR efficiency *in vivo* strongly depends on the polymerase that generates the mismatch and on the composition and location of mismatches. In one extreme case, a flanking triplet repeat sequence eliminates MMR altogether. Overall, MMR is most efficient for mismatches generated at the highest rates and having the most deleterious potential, thereby ultimately achieving high-fidelity replication of both DNA strands.

## Introduction

Three processes operate to ensure faithful replication of the eukaryotic nuclear genome [1], [2]. The first is the ability of DNA polymerases α, δ and ε to selectively insert correct rather than incorrect nucleotides onto correctly aligned rather than misaligned primer-templates. The second is proofreading, the 3′ exonucleolytic excision of errors from the primer terminus during replication. The third is mismatch repair (MMR) of errors that escape proofreading (reviewed in [3]–[7]). MMR begins when a mismatch is recognized by homologues of the bacterial MutS homodimer, either Msh2-Msh6 (MutSα) or Msh2-Msh3 (MutSβ). This recognition initiates a series of steps that ultimately remove the replication error from the nascent strand and allow new DNA to be synthesized accurately.

The origin and nature of the strand discrimination signal used for MMR *in vivo* remains uncertain. MMR requires the presence of a discontinuity in the newly synthesized strand. At least *in vitro*, this discontinuity can be a nick or gap located either 3′ or 5′ to the mismatch, with the protein requirements for MMR differing somewhat depending on the location of the DNA ends relative to the mismatch. This provides an attractive possibility (reviewed in [3]), namely that MMR may be directed to the nascent strand by the 3′ ends of growing chains at the replication fork and/or by the 5′ ends of Okazaki fragments that are transiently present during lagging strand replication. That the latter could provide a higher signal density for MMR of lagging strand replication errors was suggested in an earlier study of MMR of a damaged (8-oxo-G-A) mismatch [8]. This leads to a previously unexplored question addressed by the present study, i.e., is the efficiency of MMR similar or different for mismatches generated during leading and lagging strand replication?

Investigation of this question is complicated by the fact that DNA polymerases α, δ and ε (Pols α, δ and ε, respectively) are all required to efficiently replicate the nuclear genome [9], and these polymerases have different error rates and error specificities [2], [10]. Over the years, multiple models have been considered for the division of labor among these three polymerases during replication (reviewed in [9]–[12]). Among these models, recent evidence [2], [13], [14] suggests that under normal circumstances, the leading strand template is primarily replicated by Pol ε, while the lagging strand template is replicated by Pol α-primase and Pol δ.

Although MMR corrects errors made by all three polymerases [2], [13], [15]–[21], it has only recently become possible to determine the extent to which MMR efficiency, and possibly MMR enzymology, varies depending on the replicase that made the error, the nascent strand containing the error and/or the location of the error within a DNA strand. We are investigating these variables using *Saccharomyces cerevisiae* strains containing mutant alleles of the *POL1* (Pol α), *POL2* (Pol ε) and *POL3* (Pol δ) genes. These mutant alleles, *pol1-L868M* [18], [19], *pol2-M644G* [13] and *pol3-L612M* ([2] and references therein), encode enzymes with single animo acid replacements at the polymerase active site that reduce the fidelity of DNA synthesis. As a consequence, strains harboring these alleles have elevated spontaneous mutation rates, thereby allowing assignment of responsibility for most *in vivo* errors to a chosen mutator polymerase, rather than its wild type counterparts [2], [13]. In strains containing these mutator polymerases, *URA3* mutation rates and mutational spectra can be determined and used to calculate the rates for specific mutations, e.g., single base substitutions and insertions/deletions (indels) in various sequence contexts. Comparison of these rates in MMR-proficient yeast strains to strains that lack *MSH2*-dependent MMR yields a calculation of the apparent *MSH2*-dependent MMR efficiency for a variety of replication errors generated during replication *in vivo*.

Using this approach, we recently described the efficiency of repairing lagging strand replication errors generated by L868M Pol α and L612M Pol δ [21]. Here we extend the effort using yeast strains encoding M644G Pol ε, allowing the comparison of MMR correction efficiencies for replication errors made by each of the three eukaryotic replicative polymerases. The results indicate that on average, MMR balances the fidelity of leading and lagging strand DNA replication, but with exceptions that place some base pairs at high risk of mutation from replication infidelity even in cells with normal MMR.

## Results

The present study presents what to our knowledge is the first direct comparison of MMR efficiency for errors made by all three replicases *in vivo*, thereby providing insights into the contribution of MMR to leading and lagging strand replication fidelity. This comparison is a continuation of efforts to examine the possibility that MMR may be directed to the nascent strand by the 3′ ends of growing chains at the replication fork [22], and/or by the 5′ ends of Okazaki fragments that are transiently present during lagging strand replication [8].

### Pol ε preferentially incorporates rNMPs into the nascent leading strand

**Figure 1:**
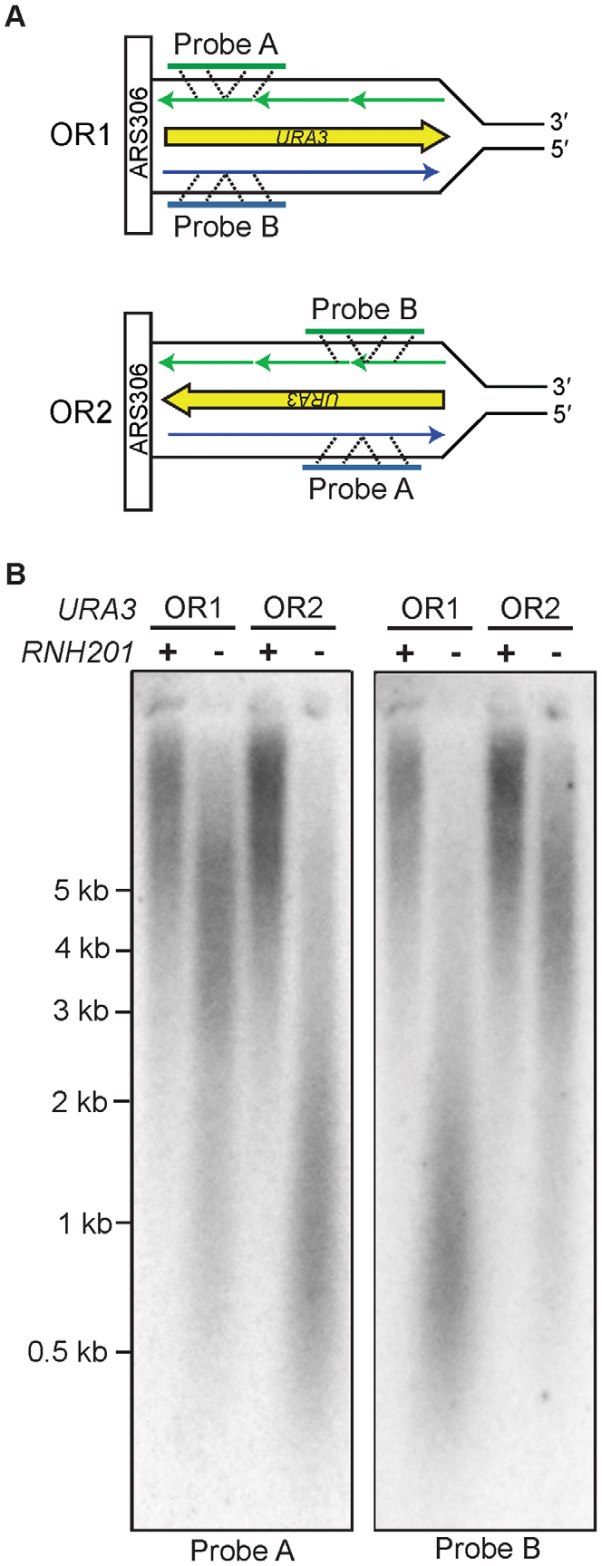
Strand-specific incorporation of rNMPs into genomic DNA. A. The orientation of the *URA3* reporter with respect to coding sequence is indicated as orientation 1 (OR1) or orientation 2 (OR2). DNA template strands are in black, the nascent leading strand is in blue and the nascent lagging strand is in green. B. Detection of alkali-sensitive sites in yeast genomic DNA reveals a strand bias for incorporation of ribonucleotides. Following alkaline hydrolysis and alkaline agarose-electrophoresis, the DNA was transferred to a nylon membrane and processed for Southern analysis. The indicated region of the *URA3* reporter gene was examined using strand-specific radiolabeled probes that anneal to either the nascent leading or nascent lagging strand. The sizes of DNA markers are indicated on the left. All strains harbor the *pol2-M644G* mutator allele. Increased DNA mobility is indicative of alkali-sensitivity due to the presence of ribonucleotides in the nascent DNA strand.

Our previous inference that Pol ε is a leading strand replicase was based on patterns of rare mutations in one gene (*URA3*) at one locus (*AGP1*) [13]. Two recent studies have made it feasible to test Pol ε strand assignment using a different biomarker, ribonucleotide incorporation into nuclear DNA. The first study demonstrated that, in addition to reduced fidelity for single base mismatches, M644G Pol ε also has reduced sugar discrimination, i.e., it incorporates rNTPs into DNA much more readily than does wild-type Pol ε [23]. In that study, rNMPs incorporated into nascent DNA during replication by M644G Pol ε were detected as alkali-sensitive sites in the nuclear genome of a *pol2-M644G rnh201Δ* strain, which lacks the ability to repair rNMPs in DNA due to deletion of the *RNH201* gene encoding the catalytic subunit of RNase H2. A more recent study exploited this fact to probe the genomic DNA of a homologous *S. pombe polε-M630F rnh201Δ* mutant strain by strand-specific Southern blotting [14]. When strand-specific probes flanking *ARS3003/3004* were used, the results revealed that more rNMPs were incorporated into the nascent leading strand than into the nascent lagging strand. This led to the interpretation that, as in budding yeast, fission yeast Pol ε is also the primary leading strand replicase [14]. Using this same strategy, we examined the strand specificity of rNMP incorporation in *S. cerevisiae pol2-M644G rnh201Δ* strains with the *URA3* reporter in one of two possible orientations, using alkali treatment and subsequent probing for either the nascent leading or lagging strand with strand-specific *URA3* probes (Figure 1A). One of the two strands from each *pol2-M644G rnh201Δ* strain was preferentially sensitive to alkaline hydrolysis (Figure 1B). In each case, this corresponded to the nascent leading strand products of replication (probe A in orientation 2 and probe B in orientation 1). These results strongly support the idea that Pol ε preferentially replicates the leading strand template. Note that the distribution of ribonucleotides within the two strands across the whole genome remains to be determined and could differ.

### Mutagenesis in MMR–proficient *pol2-M644G* strains

The strategy used here to study strand-specific MMR involves measuring spontaneous mutation rates in yeast strains with the *URA3* reporter gene present in either of two orientations, both proximal to *ARS306*, a well-characterized, early-firing replication origin [24]. In our initial study of the role of Pol ε in replication [13], we compared mutation rates in MMR proficient (*MSH2* ^+^) strains with wild type Pol ε (encoded by the *POL2* gene) to rates in strains with the *pol2-M644G* mutation. The *pol2-M644G* strains had elevated mutation rates [13], an observation that is reproduced here (Table 1). The majority of 5-FOA resistant mutants had single-base mutations in the *URA3* gene. In orientation 1, these were predominantly A-T to T-A mutations at base pairs 279 and 686. These mutations were rare in orientation 2 (partial spectra in [13], complete spectra in Figure S1A). This strong orientation bias, and the fact that the *in vitro* error rate for template T-dTMP mismatches by M644G Pol ε is much higher than the error rate for template A-dAMP mismatches, implies that Pol ε participates in leading strand DNA replication [13]. Two later studies [2], [25] indicated that Pol δ primarily acts as a lagging strand polymerase and has a less substantial role in leading strand replication. This further implied that Pol ε not only participates in leading strand DNA replication, but that it is the major leading strand replicase.

**Table 1:** Mutation rates and sequencing data for *pol2-M644G ± MSH2* strains.

*URA3* Orientation	OR1	OR2	OR1	OR2
Strain	*MSH2* a		*msh2*Δ^a^	
Mutation rate (×10^−7^)	0.18	0.21	7.2	9.5

*URA3* Orientation	OR1	OR2	OR1	OR2
Strain	*pol2-M644G MSH2* b		*pol2-M644G msh2*Δ	
Mutation rate (×10^−7^)	1.7	0.83	180	180
95% CI	1.3–2.3	0.71–0.96	120–270	130–240
*ura3* mutants sequenced	342	246	333	254
Transitions	24	36	92	83
Transversions	195	62	51	55
Single base deletions	11	9	143	71
Single base additions	1	5	19	14
Multi-base mutations	6	12	1	1

Some 5-FOA resistant mutants had no sequence change in the 804 base pair *URA3* open reading frame. These mutants were not investigated further, but they may result from epigenetic silencing, they may contain sequence changes in the promoter or 3′ untranslated region of *URA3*, or they may contain mutations in other genes that result in 5-FOA resistance.

a Expanded from [21].

b Expanded from [13].

### Mutation rates and specificity in *pol2M644G msh2Δ* strains

The *pol2-M644G msh2Δ* mutants have strongly elevated mutation rates relative to the *MSH2* ^+^ strains (Table 1), indicating that the vast majority of the mutations are made by M644G Pol ε. In the absence of mismatch repair, most 5-FOA resistant mutants contained single base changes that were widely scattered throughout the *URA3* coding sequence (Figure S1B). As compared to MMR proficient *pol2-M644G* strains, base pairs 279 and 686 in *pol2-M644G msh2Δ* strains did not stand out as hotspots for A-T to T-A transversions in orientation 1, even though base substitution and single base deletion hotspots were observed at several other locations (Figure S1B).

### MMR correction factors

The data in Table 1 and Figure S1 were used to calculate rates for single base mutations in the MMR-proficient and *msh2Δ* strains (Table S2). The ratio of these rates reflects the apparent MMR correction efficiency for each type of error, and the results can be compared (see discussion) to those reported earlier [21] for replication errors made by L868M Pol α and L612M Pol δ. As noted previously [21], [26]–[29], certain correction factors could be higher if some mismatches in the MMR proficient strains are not subject to MMR, either because they are damaged or because they are generated during DNA transactions that occur outside of replication.

**Figure 2:**
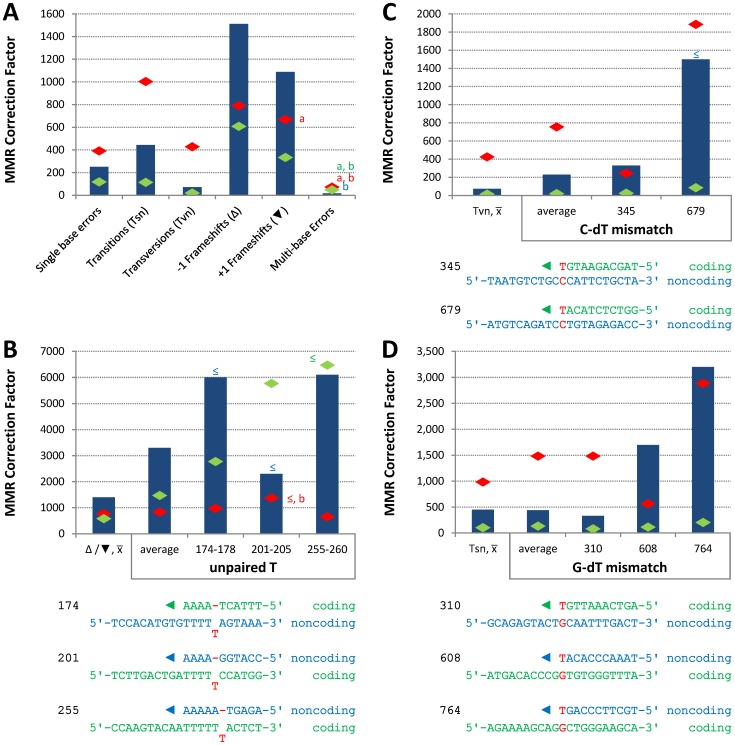
Correction factors for various mismatches made by each mutator polymerases. Mismatch repair correction factors for errors created by M644G Pol ε (blue columns), L868M Pol α (red diamonds), and L612M Pol δ (green diamonds) [21]. All correction factors are significant (p≤0.05) unless otherwise noted. (A) Correction factors for six classes of mutations across all *URA3* sequence positions. *URA3*-orientations 1 and 2 correction factors are averaged (geometric mean). Mutation class abbreviations are shown in parentheses. In panels B, C and D, for specific mutations, the inferred mismatch and the surrounding sequence context are shown below the chart. Mutation positions are shown to the left. The nascent (above) and template (below) strands are shown to the right. Triangles indicate synthesis direction. The coding strand is green, the non-coding strand is blue, and mismatched bases are red. (B) Correction factors for unpaired T bases at specific *URA3* positions, as compared to averages and general frameshift mutations. (C) Correction factors for C-dT mismatches at specific *URA3* positions, as compared to averages and general transversion mutations. Only positions 345 and 679 had sufficient observations to allow significant calculations for all three polymerases. (D) Correction factors for G-dT mismatches at specific *URA3* positions, as compared to averages and general transition mutations. *URA3* positions 310, 608, and 764 had sufficient observations. ^x–^ Average of relevant sequence positions and both *URA3* orientations. ^≤^ Upper bound, estimated by increasing *msh2Δ* observation count from 0 to 1 for purposes of correction factor calculations. ^a^ Calculated from only one *URA3* orientation due to insufficient observations in the other. ^b^ p>0.05.

Conclusions about the overall balance of repair between strands and polymerases derive from collective consideration of all single base mismatches. In the *pol2-M644G* strain background, the MMR correction factor for all single base mismatches is 250-fold (Table 2; Figure 2A, blue bar; Table S2), i.e., on average, 249 of 250 single base replication errors generated by M644G Pol ε are corrected by MMR. This correction factor is higher than for L612M Pol δ (Table 2; Figure 2A, green diamond), but lower than for L868M Pol α (Table 2; Figure 2A, red diamond). As a consequence, the mutation rates for all three variant polymerase strains are similar when MMR is operative (top line in Table 2). Average correction factors are high for each of the four classes of single base changes generated by M644G Pol ε (Figure 2A), in the following order: deletions (1,500-fold), insertions (1,100-fold), transitions (440-fold) and transversions (72-fold). Correction factors vary widely between specific positions in the *URA3* open reading frame. Figure 2B–2D show eight locations where it is possible to compare MMR of the same mismatch generated by M644G Pol ε during leading strand replication (blue bars) or by Pol α (red diamonds) and δ (green diamonds) during lagging strand replication (expanded from [21]). In order to maintain equivalent template context, leading strand errors found in one *URA3* orientation in the *pol2-M644G* strains are always compared to lagging strand errors found in the other *URA3* orientation in the *pol1-L868M* and *pol3-L612M* strains. For example, the correction factors in Figure 2B for deleting an A-T pair from the three longest runs of A-T pairs in the *URA3* coding sequence (base pairs 174–178, 201–205 and 255–260, Figure S1) are each inferred to involve a single unpaired T. The comparative MMR correction factors and their implications are considered in the Discussion.

**Table 2:** Mutation rates and correction factors for all single-base mismatches in three mutator polymerase backgrounds.

	*pol2-M644G*	*pol1-L868M* b	*pol3-L612M* b
*MSH2* rate	6.5×10^−8^ a	7.0×10^−8^	1.4×10^−7^
*msh2Δ* rate	1.6×10^−5^	5.2×10^−5^	2.2×10^−5^
MMR efficiency	250×	740×	160×

a Expanded from [13].

b Expanded from [21].

### A-T to T-A transversions at base pair 686

**Figure 3:**
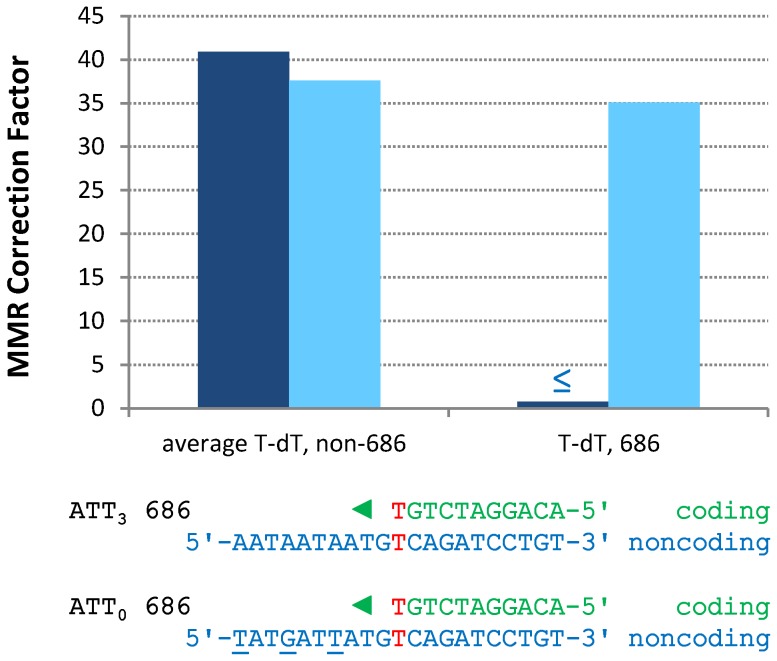
Restoration of T-dT repair at position 686 by removing a flanking triplet repeat. Mismatch repair correction factors for errors created by M644G Pol ε in *ATT_3_* (dark blue columns) and *ATT_0_* (light blue columns) *URA3* sequences. The T-dT mismatch at position 686 and the surrounding sequence context are shown below the chart: nascent strand above; template strand below. Triangles indicate the direction of synthesis. The three silent mutations made to convert *ATT_3_* into *ATT_0_ URA3* are underlined. Note that the polymerase active site encounters the triplet repeat in the *ATT_3_* template strand *after* making the T-dT mismatch at position 686. ^≤^ Upper bound, estimated by increasing *msh2Δ* observation count from 0 to 1 for purposes of correction factor calculations.

In contrast to the efficient repair of most single mismatches, the rate of A-T to T-A transversions at base pair 686 in orientation 1 (Figure S1 and Table S3) is no higher in the *pol2-M644G msh2Δ* strain than in the MMR-proficient *pol2-M644G* strain (Table S2). This indicates that T-T mismatches generated at base pair 686 during leading strand replication by M644G Pol ε are not efficiently corrected by MMR (Figure 3, “T-dT, 686,” dark blue bar). This contrasts with an average of 41-fold correction (dark blue bar on left) of the same mismatch inferred at all other A-T base pairs in *URA3*, i.e., A to T substitutions in orientation 1 and T to A transversions in orientation 2 (Figure S1). Adjacent to base pair 686 is a triplet repeat sequence, 5′-ATT ATT ATT gTT (designated here as ATT_3_). For several reasons (see Discussion), we speculated that this sequence might suppress MMR at base pair 686. To test this, we constructed strains in which ATT_3_ was modified to 5′-ATA ATC ATA gTT (designated ATT_0_, see Figure 3), with the three (underlined) changes interrupting the repeat units without changing the amino acid sequence. We then measured spontaneous mutation rates and generated mutational spectra (Figure S2) to determine if the flanking sequence changes allowed MMR of T-T mismatches at base pair 686. The results (Table S3) indicate that this is indeed the case. The MMR correction factor at base pair 686 increased to 35-fold (Figure 3, p≤0.001), indicating that 97% of T-T mismatches are repaired when base pair 686 is flanked by ATT_0_.

Single base-base mismatches are repaired by MutSα (Msh2-Msh6) but not by MutSβ (Msh2-Msh3) [3]–[7], implying that the ATT_3_ sequence is suppressing repair of the T-T mismatch that would normally occur via MutSα. However, given evidence that MutSβ can bind to a non-B-DNA structure that can form in a triplet repeat sequence and promote triplet repeat expansion (reviewed [30]), we examined whether suppression of MMR at base pair 686 might depend on MutSβ. This was done by calculating the A-T to T-A mutation rate at base pair 686 in *URA3* orientation 1 in a *pol2-M644G msh3Δ rnh201Δ* strain [31]. The calculated A-T to T-A rate is 17×10^−8^, which is no lower than observed here in the Msh3^+^ strain (6.8×10^−8^, Table S3). Thus suppression of MMR by ATT_3_ is independent of MutSβ.

## Discussion

This study provides new insights into relationships between the intrinsic asymmetry of DNA replication and MMR in yeast.

### Ribonucleotides are “biomarkers” of Pol ε action *in vivo*

We previously inferred that Pol ε participates in leading strand replication using base substitutions as biomarkers for leading strand replication. These events are rare, occurring approximately once per 10 million incorporations. The present study uses ribonucleotides as an independent and much more abundant biomarker. The preferential presence of ribonucleotides in the nascent leading strand observed here in *pol2-M644G rnh201Δ* strains (*URA3* orientation 1 and orientation 2; Figure 1) strongly supports the inference that Pol ε primarily participates in leading strand replication. This does not preclude occasional Pol ε participation in lagging strand replication. The interpretation that Pol ε primarily participates in leading strand replication lends credibility to the interpretations presented below regarding the efficiency of repairing mismatches made by Pol ε during leading strand replication as compared to mismatches of similar composition made by Pols α and δ during lagging strand replication. An additional notable point here is that the sizes of the nascent leading strand fragments resulting from alkaline hydrolysis of DNA from the *pol2-M644G rnh201Δ* strains (Figure 1) indicate that approximately one ribonucleotide may be incorporated for every 1,000 deoxyribonucleotides. This density of ribonucleotide incorporation into DNA is about four orders of magnitude higher than for A-T- to T-A transversions. Thus ribonucleotides mapped by deep sequencing techniques could serve as high density, genome-wide biomarkers of Pol ε action *in vivo* during replication and possibly during repair and recombination.

### Variations in repairing M644G Pol ε replication errors

The average MMR correction factors for errors made by M644G Pol ε are highest for indels, intermediate for transitions and lowest for transversions (Figure 2A). This rank order is common to *E. coli* [28], [29], [32] and to errors made by yeast Pols α and δ [21], suggesting that MMR has conserved the ability to most efficiently correct the most potentially deleterious errors (indels), and also the base-base mismatches made at the highest rates by both bacterial and eukaryotic replicases. This general principal is qualified by the observation that MMR efficiency varies, even for the same inferred mismatch (e.g., either an extra T, a C-dT or a G-dT mismatch, Figure 2B, 2C and 2D, respectively) made by the same polymerase (M644G Pol ε) during replication of the same (leading) strand. Most sequence-dependent variations in MMR efficiency seen here are in the 2- to 10-fold range (Figure 2) depending on the comparison. That such variations are typically small is perhaps expected, since MMR is needed to preserve the stability of nuclear genomes despite their enormous sequence complexity.

Variations due to mismatch composition and location are consistent with biochemical studies showing differences in MMR *in vitro* [33] and with mutational studies *in vivo* in which the identity of the replicase that made the mismatch was unknown. Several explanations for variations in eukaryotic MMR efficiency can be explored in the future. For example, the efficiency with which *E. coli* repairs transversion mismatches in phage λ increases with increasing G-C content in neighboring nucleotides [32], and recognition of certain mismatches by MutSα is influenced by a 6-nucleotide region surrounding the mismatch [34]. Thus it may be that flanking sequences, such as those shown in Figure 2, influence eukaryotic MMR efficiency *in vivo* by modulating (i) mismatch binding by MutSα, which contacts several base pairs on either side of the mismatch [35], (ii) base pair stacking, since a MutSα-bound mismatched base stacks with a conserved phenylalanine in Msh6, and/or (iii) DNA flexibility, since MutSα-bound mismatched DNA is kinked, and a transition between bent and unbent DNA may be critical for limiting MMR to processing of mismatched as compared to matched base pairs [36]. Variations in MMR efficiency might also depend on proteins that operate downstream of mismatch binding, such as MutLα or exonucleases, or they may reflect other variables, such as the timing of nucleosome reloading behind the replication fork, nucleosome dynamics and/or chromatin remodeling.

### A natural DNA sequence that suppresses MMR

A striking observation here is the apparent absence of MMR of the A-T to T-A transversion at base pair 686 (Figure 3), which is inferred to result from a T-T mismatch made by M644G Pol ε during leading strand replication. This lack of repair contrasts sharply with efficient repair at many other locations. For example, the deletion mismatch at base pairs 255–260, which is predicted to involve a mismatch containing a single unpaired T in the template (Figure 2B), has an approximately 6000-fold higher correction factor than for the T-T mismatch at base pair 686. Lack of repair at base pair 686 is not due to a general inability to correct A-T to T-A transversion mismatches, because the average correction factor for these events elsewhere in *URA3* is 41-fold (Figure 3). The absence of correction at position 686 led us to test whether MMR was inhibited by the adjacent 5′-ATTATTATTgTT sequence. There were several reasons to suspect that this could be the case. The sequence is A-T rich and may have unusual helical parameters that could diminish MMR. For example, sequences containing larger numbers of ATT repeats can form a non-hydrogen bonded structure [37], and can be induced into hairpins by the DNA minor groove binding ligand DAPI (4′,6-diamidino-2-phenylindole) [38], [39]. Triplet repeat sequences can form non-B-DNA structures that bind MMR proteins (reviewed in [30]), and they are often associated with genome instability (reviewed in [40]), albeit characterized by indels rather than base substitutions. In addition, recent studies have demonstrated that nucleosomes influence the behavior of MMR proteins and visa versa (e.g., see [41]–[44]), and nucleosome binding to DNA is influenced by DNA sequence, with A-T-rich dinucleotides such as those present in ATT_3_ having an important role in nucleosome positioning (e.g., see [45], [46] and references therein).

For these reasons, we examined MMR at base pair 686 after changing the flanking sequence to eliminate the triplet repeats and decrease A-T content by one base pair. The results indicate that these changes allowed correction of 97% of the mismatches generated by M644G Pol ε at base pair 686 (88% correction at the lower 95% confidence limit, Figure 3). This suggests that the ATT_3_ flanking sequence is a natural *cis*-acting suppressor of the normal MSH2-dependent MMR machinery. Suppression does not decrease upon deletion of *MSH3*, and thus is MutSβ independent, unlike triplet repeat expansion [30]. Collectively, position 686 and ATT_3_ are an example of what has been called an “At Risk sequence Motif” [47], i.e., a naturally occurring DNA sequence that results in inefficient operation of a DNA transaction required for genome stability. The fact that one such sequence exists in the 804 base pair open reading frame of *URA3* leads one to wonder how many natural suppressors of MMR might be present in nuclear genomes. This issue is currently being investigated using the deep sequencing approach previously used to infer that Pol δ is a lagging strand replicase across the yeast genome [25]. Experiments are also planned to examine which (if any) of the possibilities mentioned in the preceding section may be relevant to inefficient MMR at base pair 686.

### Correcting leading and lagging strand replication errors

We previously suggested that MMR may be directed to the nascent strand by the 3′ ends of growing chains at the replication fork [22], and/or by the 5′ ends of Okazaki fragments that are transiently present during lagging strand replication [8]. The 5′ ends of Okazaki fragments, and perhaps the PCNA required to process these ends, could potentially provide a higher signal density for MMR of lagging strand replication errors as compared to errors generated during leading strand replication, which is thought to be more continuous. If so, then MMR might be more efficient in correcting lagging strand errors. In an initial test of this hypothesis, we found that mutagenesis due to a mismatch formed at one particular G-C base pair during replication of unrepaired 8-oxo-G in *ogg1*-deficient yeast was lower for lagging as compared to leading strand replication, and importantly, that this bias was largely eliminated in MMR defective strains [8]. Among several possible explanations that we considered for loss of the strand bias, one was that 8-oxo-G-dA mismatches made during lagging strand replication may be more efficiently corrected than are 8-oxo-G-dA mismatches made during leading strand replication. A major goal of the present study was to test this hypothesis for multiple, natural (i.e., undamaged) mismatches generated at different locations during replication of a larger target sequence. The present study accomplishes this, and allows the first direct comparison of MMR efficiency for errors made by all three replicases, to our knowledge, thereby providing insights into the contribution of MMR to leading and lagging strand replication fidelity.

From the results in Figure 2, we conclude that in general, mismatches made by all three replicases are repaired very efficiently. This is logical given the need to preserve genetic information in both DNA strands. This conclusion is independent of various models regarding which DNA polymerase replicates which strand (reviewed in [11], [12]). Other implications derive from the model wherein Pols α and δ are the primary lagging strand replicases and Pol ε is the primary leading strand replicase. In our earlier report [21], we pointed out that correction factors were higher for mismatches made by Pol α than for the same mismatches made by Pol δ, suggesting that the 5′ ends of Okazaki fragments may be strand discrimination signals and that MMR efficiency may be related to the proximity of a mismatch to that signal. This is interesting given that DNA polymerase ε is highly processive, at least as processive as DNA polymerase δ, and that leading strand replication is thought to be largely continuous [48], [49], [50]. It is of course conceivable that leading strand replication may not be as continuous as current models imply. If leading strand replication is indeed largely continuous, then the fact that MMR corrects most Pol ε errors about as efficiently as it corrects errors made by Pols α and δ (Figure 2) implies the existence of MMR signals other than the 5′ ends of Okazaki fragments, and these can very efficiently direct MMR to the nascent leading strand. Possible signals for leading strand replication include the above-mentioned 3′ ends of growing chains at the replication fork [22], [51], [52], nicks introduced into the nascent leading strand by nucleases, and/or asymmetrically bound PCNA [8], [53]. PCNA is a particularly attractive possibility for differentially modulating the efficiency of MMR of errors made by the three replicases, because it is involved in early steps in MMR (see [3]–[7] for review]), it does not influence DNA synthesis by Pol α, and it does stimulate DNA synthesis by both Pol δ and Pol ε, albeit through different PCNA-polymerase interactions (see [9] and references therein).

The results in Figure 2 further suggest that, even for the same mismatch (extra T, G-dT or C-dT) in a common sequence context, MMR efficiency varies depending on which polymerase made the error. In two of three instances involving deletion of a single template T (Figure 2B), the repair of mismatches made by Pol δ is higher than for mismatches made by Pol ε. This correlates with the observation that Pol δ generates this mismatch *in vitro* at a higher rate than does Pol ε [54]. Similarly, transitions and transversions (Figure 2A) and several site-specific base substitutions (Figure 2C and 2D) generated by Pol α are corrected more efficiently than are mismatches generated by Pol δ and Pol ε. Pol α lacks an intrinsic proofreading exonuclease activity and is less accurate than proofreading-proficient Pols δ and ε (reviewed in [10], [55]). Thus the present study of mismatches generated by Pol ε extends the idea that MMR has evolved to most efficiently correct the most deleterious mismatches (i.e., indel mismatches). Within classes of similar deleterious potential (base-base mismatches), evolution has produced the highest efficiency versus the most frequently generated mismatches. In a model wherein Pol ε is the major leading strand replicase and Pols α and δ conduct about 10% and 90% of lagging strand replication [2], respectively, the results (Table 2; Figure 2A, average repair for single base errors) further suggest that MMR balances the fidelity of replication of the two strands despite the use of replicases with substantially different fidelity and error specificity.

## Materials and Methods

### Strains, mutation rates, and analysis of *ura3* mutants

The strains used in this study, the measurements of spontaneous mutation rates and the sequencing of *URA3* mutants were as previously described [2], [13], [21], save that *MSH2* was deleted from haploid *pol2-M644G* strains rather than diploid. The ATT_3_ to ATT_0_ conversion was made via site-directed mutagenesis and integration pop-out [56] in a strain with wild type polymerases. PCR product containing the ATT_0_ *URA3* allele was then transformed into *msh2Δ* backgrounds and proper insertion verified via sequencing.

### Probing for alkali-sensitive sites in genomic DNA

Genomic DNA was isolated from exponentially growing cultures (grown in YPDA at 30°C) using the Epicentre Yeast DNA purification kit. Five µg of DNA was treated with 0.3 M KOH for 2 h at 55°C and subjected to alkaline-agarose electrophoresis as described [23]. Following neutralization, DNA was transferred to a charged nylon membrane (Hybond N+) by capillary action and probed by Southern analysis. Strand-specific radiolabeled probes were prepared from a PCR-amplified fragment of *URA3* template, using a previously described procedure and probe design [14].

### Statistical analysis

See Text S1.

## Supporting Information

Figure S1 Mutational spectra in *pol2-M644G and pol2-M644G msh2Δ* strains. The *URA3* reporter was present in either orientation 1 (OR1) or orientation 2 (OR2) at position *AGP1*. The coding strand of the *URA3* open reading frame is shown, with every 10^th^ base indicated by a dot and mutations depicted above (OR1) and below (OR2) the wild type *URA3* sequence. Single letters represent base substitutions, open triangles represent single base deletions, and closed triangles represent single base additions. Indels in homonucleotide runs are shown at the 5′-most position of the run. (*A*) Spectra in *MSH2* strains [13]. (*B*) Spectra in *msh2Δ* strains.
(TIF)

Figure S2 Mutational spectra in *pol2-M644G* and *pol2-M644G msh2Δ* strains with *ATT_0_ URA3*. As for Figure S1, with spectra for the *pol2-M644G msh2Δ* and *pol2M644G MSH2* strains shown above and below the ATT_0_ *URA3* sequence, respectively. The three bases that differ between the ATT_3_ and ATT_0_ *URA3* sequences are shown in bold (positions 690, 693, and 696).
(TIF)

Table S1 Multi-base mutations omitted from spectrum figures.
(DOCX)

Table S2 Mutation counts, mutation rates, and MMR correction factors.
(XLSX)

Table S3 Mutation Rates for *pol2-M644G ± msh2Δ* and *ATT_3_/ATT_0_ URA3 Orientation 1* strains. ^a^ Expanded from [21]. ^b^ Expanded from [13].
(DOCX)

Text S1 The supporting information includes methods for calculating and statistical analyses of mutation rates and correction factors.
(DOC)
