# Supplementary figures and images for: Mismatch Repair Balances Leading and Lagging Strand DNA Replication Fidelity
Source: PLoS Genet. 2012 Oct 11;8(10):e1003016. doi: 10.1371/journal.pgen.1003016 (PMC3469411; doi:10.1371/journal.pgen.1003016)

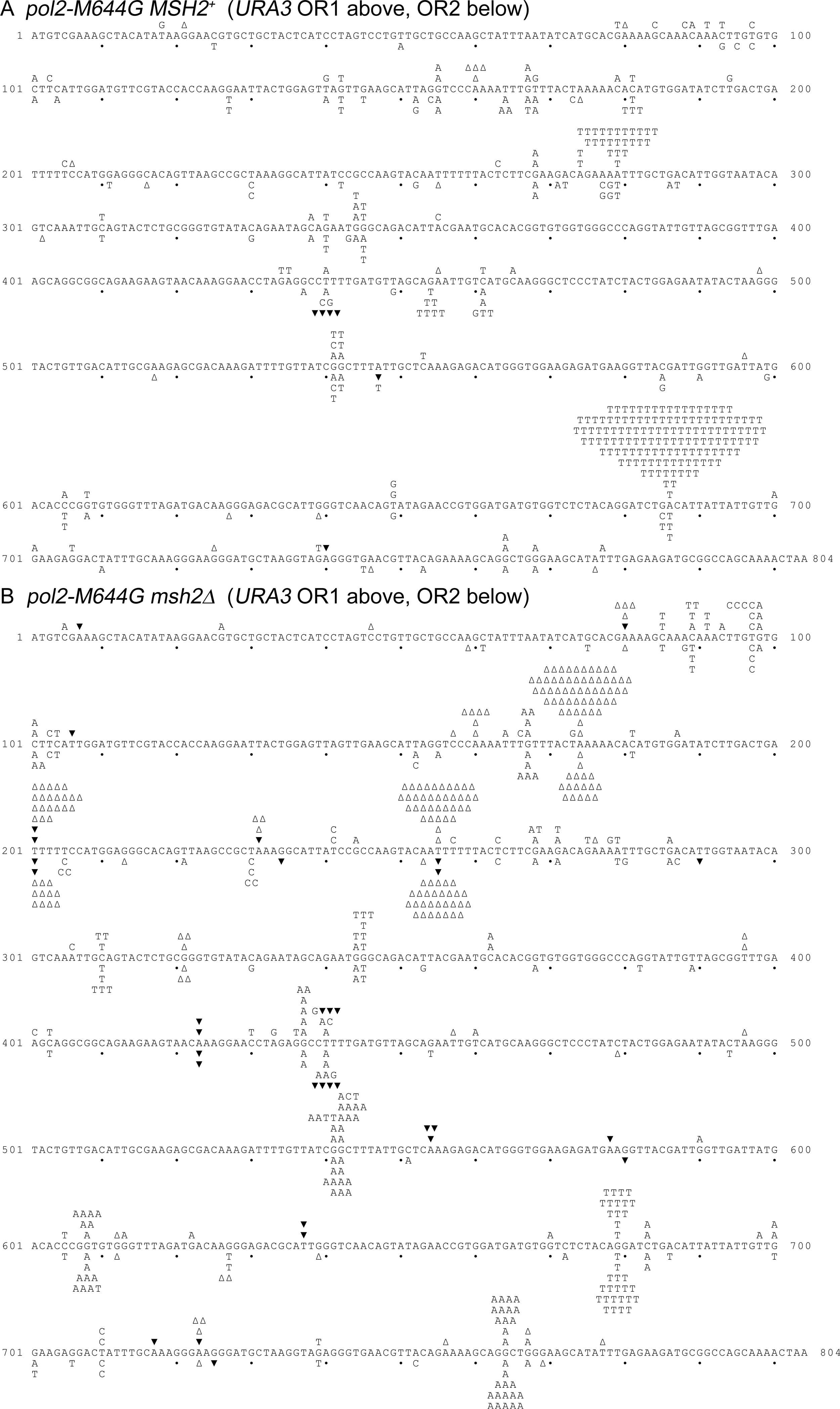

Supplement: Figure S1 — Mutational spectra in pol2-M644G and pol2-M644G msh2Δ strains. The URA3 reporter was present in either orientation 1 (OR1) or orientation 2 (OR2) at position AGP1. The coding strand of the URA3 open reading frame is shown, with every 10th base indicated by a dot and mutations depicted above (OR1) and below (OR2) the wild type URA3 sequence. Single letters represent base substitutions, open triangles represent single base deletions, and closed triangles represent single base additions. Indels in homonucleotide runs are shown at the 5′-most position of the run. (A) Spectra in MSH2 strains [13]. (B) Spectra in msh2Δ strains. (TIF) [file pgen.1003016.s001.tif]

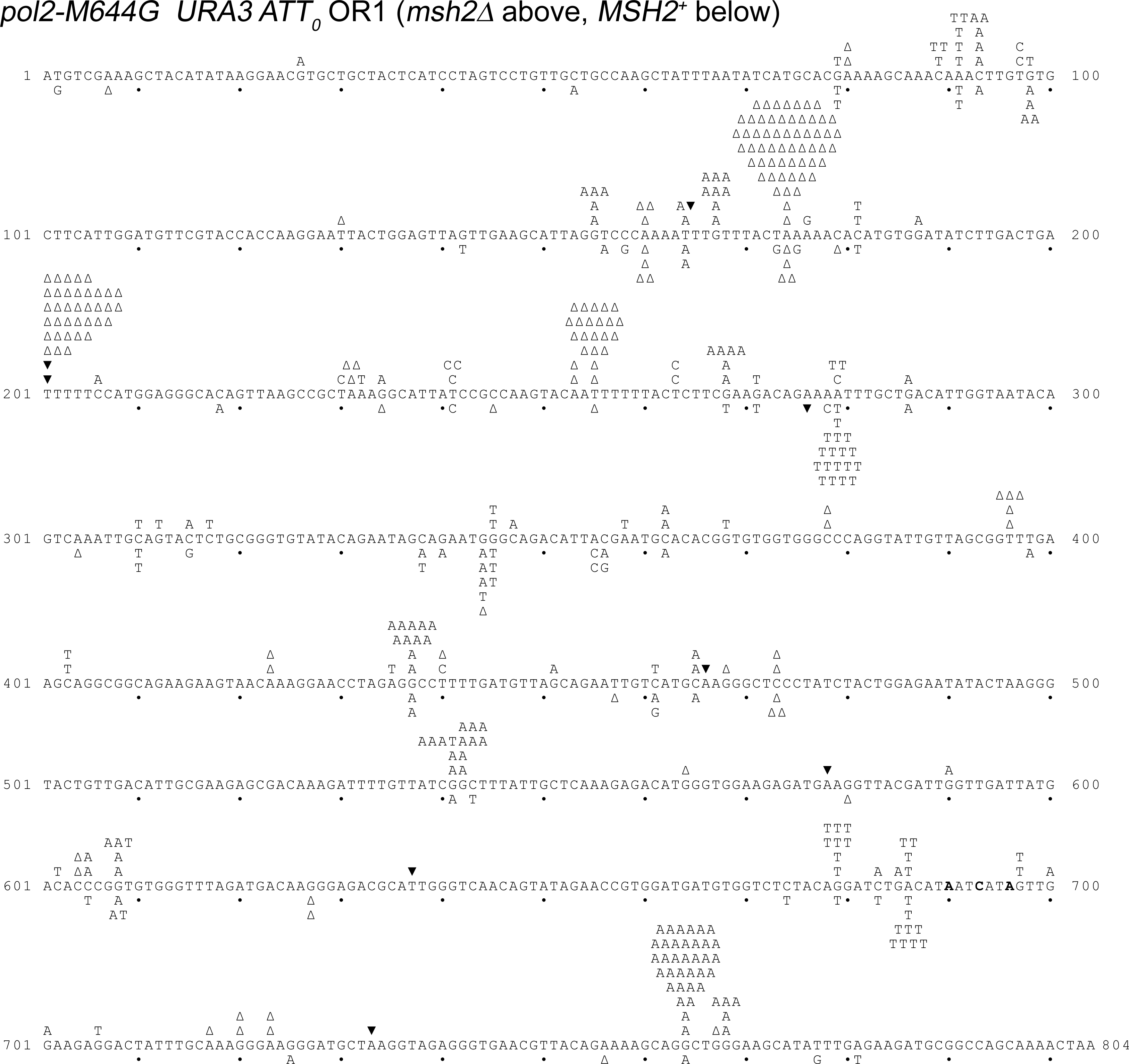

Supplement: Figure S2 — Mutational spectra in pol2-M644G and pol2-M644G msh2Δ strains with ATT0 URA3. As for Figure S1, with spectra for the pol2-M644G msh2Δ and pol2M644G MSH2 strains shown above and below the ATT0 URA3 sequence, respectively. The three bases that differ between the ATT3 and ATT0 URA3 sequences are shown in bold (positions 690, 693, and 696). (TIF) [file pgen.1003016.s002.tif]
