# Supplementary material for: Mismatch Repair Balances Leading and Lagging Strand DNA Replication Fidelity
Source: PLoS Genet. 2012 Oct 11;8(10):e1003016. doi: 10.1371/journal.pgen.1003016 (PMC3469411; doi:10.1371/journal.pgen.1003016)
Supplement: Table S1 — Multi-base mutations omitted from spectrum figures. (DOCX) [file pgen.1003016.s003.docx]

**Table S1.**

| **Strain** | **Count** | **Size**  **(bp)** | **Start**  **(bp)** | **End**  **(bp)** | **Mutation type** | **Notes** |
| --- | --- | --- | --- | --- | --- | --- |
| *pol2-M644G URA3 OR1* | 1 | 2 | 85 | 86 | tandem duplication |  |
| *pol2-M644G URA3 OR1* | 1 | 63 | 286 | 348 | deletion |  |
| *pol2-M644G URA3 OR1* | 1 | 8 | 430 | 437 | deletion |  |
| *pol2-M644G URA3 OR1* | 2 | 3 | 688 | 690 | deletion |  |
| *pol2-M644G URA3 OR2* | 4 | 24 | 27 | 50 | deletion |  |
| *pol2-M644G URA3 OR2* | 1 | 3 | 118 | 120 | deletion |  |
| *pol2-M644G URA3 OR2* | 1 | 53 | 260 | 312 | deletion |  |
| *pol2-M644G URA3 OR2* | 1 | 57 | 272 | 328 | tandem duplication |  |
| *pol2-M644G URA3 OR2* | 1 | 27 | 366 | 392 | tandem duplication |  |
| *pol2-M644G URA3 OR2* | 1 | 4 | 501 | 504 | tandem duplication |  |
| *pol2-M644G URA3 OR2* | 2 | 2 | 554 | 555 | deletion |  |
| *pol2-M644G URA3 OR2* | 1 | 2 | 777 | 778 | tandem base pair substitution | TT to AG |
| *pol2-M644G msh2Δ URA3 OR1* | 1 | 2 | 201 | 202 | deletion |  |
| *pol2-M644G msh2Δ URA3 OR2* | 1 | 3 | 688 | 690 | deletion |  |
| *pol2-M644G ATT0 URA3 OR1* | 1 | 3 | 86 | 88 | complex mutation | AAA to CAC |
| *pol2-M644G ATT0 URA3 OR1* | 1 | 6 | 222 | 227 | insertion | TTTATA |
| *pol2-M644G ATT0 URA3 OR1* | 1 | 3 | 237 | 239 | deletion |  |
| *pol2-M644G ATT0 URA3 OR1* | 1 | 17 | 590 | 606 | tandem duplication |  |
| *pol2-M644G ATT0 URA3 OR1* | 1 | 6 | 615 | 620 | complex mutation | TTTAG to TTTTAT |
| *pol2-M644G ATT0 URA3 OR1* | 1 | 47 | 720 | 766 | tandem duplication |  |
| *pol2-M644G msh2Δ ATT0 URA3 OR1* | 1 | 8 | 330 | 337 | deletion |  |
| *pol2-M644G msh2Δ ATT0 URA3 OR1* | 1 | 13 | 404 | 416 | tandem duplication |  |
