# Supplementary material for: Mismatch Repair Balances Leading and Lagging Strand DNA Replication Fidelity
Source: PLoS Genet. 2012 Oct 11;8(10):e1003016. doi: 10.1371/journal.pgen.1003016 (PMC3469411; doi:10.1371/journal.pgen.1003016)
Supplement: Table S3 — Mutation Rates for pol2-M644G ± msh2Δ and ATT3/ATT0 URA3 Orientation 1 strains. a Expanded from [21]. b Expanded from [13]. (DOCX) [file pgen.1003016.s005.docx]

**Table S3.**

|  | ***MSH2*** ^a^ | ***msh2∆*** ^a^ | **cf** | ***MSH2*** ^b^ | ***msh2∆*** | **cf** |
| --- | --- | --- | --- | --- | --- | --- |
| **ATT_3_ Strains** | ***Wild type pols*** | |  | ***pol2-M644G*** | |  |
| Mutation rate (x 10^-7^) | 0.18 | 7.2 |  | 1.7 | 180 |  |
| 95% CI | 0.14-0.23 | 3.5-15 |  | 1.3-2.3 | 120-270 |  |
| A to T at 279 (x 10^-7^) | 0.0034 | ≤ 0.037 | ≤ 11x | 0.12 | 0.54 | 4.5x |
| A to T at 686 (x 10^-7^) | 0.0017 | ≤ 0.037 | ≤ 22x | 0.68 | ≤ 0.54 | ≤ 0.78x |
| **ATT_0_ Strains** | ***Wild type pols*** | |  | ***pol2-M644G*** | |  |
| Mutation rate (x 10^-7^) | 0.67 | 7.6 |  | 1.3 | 170 |  |
| 95% CI | 0.50-0.90 | 5.9-9.8 |  | 0.87-1.8 | 100-280 |  |
| A to T at 279 (x 10^-7^) | 0.0086 | ≤ 0.083 | ≤ 9.7x | 0.11 | 0.93 | 8.4x |
| A to T at 686 (x 10^-7^) | 0.0043 | ≤ 0.083 | ≤ 20x | 0.053 | 1.9 | 35x |
