# Supplementary material for: Mismatch Repair Balances Leading and Lagging Strand DNA Replication Fidelity
Source: PLoS Genet. 2012 Oct 11;8(10):e1003016. doi: 10.1371/journal.pgen.1003016 (PMC3469411; doi:10.1371/journal.pgen.1003016)
Supplement: Text S1 — The supporting information includes methods for calculating and statistical analyses of mutation rates and correction factors. (DOC) [file pgen.1003016.s006.doc]

**Supplemental Experimental Procedures**

**Calculation of mutation rates and correction factors.** *URA3* spontaneous mutation rates (collected as per Nick McElhinny et al., 2008; Pursell et al., 2007) for any given strain were estimated from mutation frequencies. Mutation rates for any given mutation type (subset) within any given mutation spectrum were calculated as follows:

Eq. 1
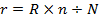
 ,

where *R* is the geometric mean overall *URA3* mutation rate, *r* is the mutation rate for a subset of mutations within the given spectrum (e.g. A to T transversions or +1 base frameshifts), *N* is the total number of *URA3* sequences collected (not the total number of mutations found), and *n* is the number of mutations in the chosen subset.

Mismatch repair (MMR) correction factors (cf's) for each chosen subset of mutations were estimated by comparing the mutation rates for that subset from mismatch repair proficient (*MSH2^+^*) and deficient (*msh2Δ*) as follows:

Eq. 2
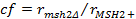
 .

**Statistical analyses of mutation rates and correction factors.** Mutation rates from fluctuation experiments are distributed as bell-shaped curves in the logarithmic domain. Though the discreet nature of mutation events would suggest a log-binomial distribution, these curves were approximated as log-normal distributions for ease of analysis (e.g confidence intervals, p-values).

Two sets of p-values are quoted herein. The first is for comparing correction factors to unity in order to prove significant repair (see main text pertaining to Figures 2 and 3). The second is for comparing correction factors to each other in order to prove differences in repair (see main text pertaining to Figure 3). P-values were estimated as the proportion of at least 1000 Monte Carlo simulations exceeding the relevant comparators. Overall rates (*R* in Eq. 1) were simulated with log-normal distributions as calculated from fluctuation experiments. Mutation counts (*n* in Eq. 1) were simulated assuming binomial distributions with the number of trials equal to the overall number of mutations within the spectrum (*n_T_*), probability equal to the observes observation frequency within the spectrum (*n/n_T_*), and the criterion value set to a random number selected from a uniform distribution between 0 and 1.
